# Reply to Brzozek et al. Comment on “Choi et al. Cellular Phone Use and Risk of Tumors: Systematic Review and Meta-Analysis. *Int. J. Environ. Res. Public Health* 2020, *17*, 8079”

**DOI:** 10.3390/ijerph18115581

**Published:** 2021-05-24

**Authors:** Joel M. Moskowitz, Seung-Kwon Myung, Yoon-Jung Choi, Yun-Chul Hong

**Affiliations:** 1 School of Public Health, University of California, Berkeley, CA 94720, USA; 2 Department of Cancer Biomedical Science, National Cancer Center Graduate School of Cancer Science and Policy, Goyang 10408, Korea; 3 National Cancer Center, Department of Family Medicine and Center for Cancer Prevention and Detection, Hospital, Goyang 10408, Korea; 4 Division of Cancer Epidemiology and Management, National Cancer Center Research Institute, Goyang 10408, Korea; 5 Department of Preventive Medicine, College of Medicine, Seoul National University, Seoul 110-744, Korea; hierica8@snu.ac.kr (Y.-J.C.); ychong1@snu.ac.kr (Y.-C.H.); 6 Environmental Health Center, College of Medicine, Seoul National University, Seoul 03080, Korea; 7 Institute of Environmental Medicine, Seoul National University Medical Research Center, Seoul 03080, Korea

We appreciate Christopher Brzozek and his colleagues’ interest [1] in our study [2] and their recognition of the importance of our latest systematic review of the research regarding tumor risk from mobile phone (i.e., cell phone) use. This paper updated the research we reviewed in our 2009 meta-analysis [3].

The Brzozek et al. letter is the second letter to the editor regarding our current study. Ken Karipidis [1] and Martin Röösli [4], the senior authors of these two letters, are two of the 14 commissioners on the International Commission on Non-Ionizing Radiation Protection (ICNIRP). Their interest in our paper is not likely coincidental, because the major finding from both of our review studies was that heavier, long-term cell phone use was associated with significantly increased tumor risk [2,3]. Because this finding calls into question the adequacy of ICNIRP’s radio frequency exposure guidelines to protect human health, ICNIRP may have a vested interest in manufacturing doubt about our research.

Since all but one of the concerns in the Brzozek letter were raised in the letter by de Vocht and Röösli [4], to avoid redundancy, we request that readers see our response to de Vocht and Röösli which addresses these other concerns [5]. Hence, in our reply below, we simply address Brzozek et al.’s unique concern about our paper.

Brzozek et al.’s [1] first concern is that we “unfairly and repeatedly criticized” the INTERPHONE group [6] and many of its investigators for their reliance on cellular phone industry funding. They further criticized us for providing only one reference [7] about possible “’hidden conflicts’ of interest” among the INTERPHONE investigators.

Industry-funded research, especially occupational and environmental health research, has been a topic of concern for decades, because it has often been found to be biased to support industry interests, according to numerous peer-reviewed papers, e.g., [8,9,10,11,12,13,14,15,16,17,18,19]. Moreover, several peer-reviewed papers have raised concerns about bias in industry-funded research on the effects of exposure to radio frequency radiation [20,21,22,23,24].

Multiple papers that reviewed the research on the effects of radio frequency or cell phone radiation have found that industry-funded studies were less likely to report effects [3,25,26,27,28,29,30,31]. For example, in 2007, a review of experimental studies conducted by Martin Röösli and colleagues concluded, “The interpretation of results from studies of health effects of radiofrequency radiation should take sponsorship into account” [25]. In an updated review published three years later they concluded, “Previous findings were confirmed: industry-sponsored studies were least likely to report results suggesting effects… The source of funding and conflicts of interest are important to consider in this area of research” [27]. We are aware of only one review study of the effects of radio frequency radiation that failed to find an association between study outcomes and study sponsorship [32].

We believe our discussion of the INTERPHONE study group is balanced and fair. Although our paper mentioned industry funding of the INTERPHONE study three times, these mentions amounted to about 200 words in our 5000-word manuscript. Twice when we discussed this issue, we provided a potential methodological explanation (i.e., selection bias) for INTERPHONE’s anomalous results. Although the INTERPHONE study found significantly increased tumor incidence among those who were the heaviest cell phone users, the study reported significantly decreased tumor incidence among “regular” cell phone users [6].

In the third instance when we [2] cited Hardell and Carlberg‘s concern [7] regarding potential “’hidden conflicts’ of interest” among INTERPHONE investigators, we pointed out that the “authors reported that the provision of funds to the study investigators via the UICC was governed by agreements that guaranteed INTERPHONE’s complete scientific independence” [2].

The following are the three instances in our paper [2] that addressed the issue of INTERPHONE’s funding:

“All of the INTERPHONE studies were partly funded by the cellular phone industry (precisely, supported by funding from the International Union against Cancer, which received funds from the Mobile Manufacturers’ Forum and Global System for Mobile Communications Association) except for the INTERPHONE-Japan studies.”

“Perhaps due to methodological deficiencies, cellular phone use appeared to reduce tumor risk in the INTERPHONE studies. These studies were partly funded by the mobile industry, had poor methodological quality, showed larger differences in response rates between the case and control groups, and did not use blinding at interview.”

“Thus, the decreased risks of tumors observed in the INTERPHONE studies might be due to selection bias from participation of cellular phone users in the control group [69]. We also found that studies partly funded by the cellular phone industry showed a statistically significantly decreased risk of tumors by cellular phone use, all of which were INTERPHONE studies. It remains unclear whether cellular phone industry funding affected the study planning and conduct or data analysis and interpretation because the authors reported that the provision of funds to the study investigators via the UICC was governed by agreements that guaranteed INTERPHONE’s complete scientific independence. Nonetheless, many of these investigators rely upon industry for future research funding so they may have “hidden conflicts” of interest despite such agreements [70].”
